# ADDRESSING CO-OCCURRING ALCOHOL AND MENTAL HEALTH PROBLEMS IN OLDER ADULTS: CO-DESIGNED DIRECTIONS FOR PRACTICE

**DOI:** 10.1093/geroni/igad104.1871

**Published:** 2023-12-21

**Authors:** Bethany Bareham, Deepti John, Amy O’Donnell, Jennifer Liddle, Barbara Hanratty

**Affiliations:** Newcastle University, Newcastle upon Tyne, England, United Kingdom; Newcastle University, Newcastle Upon Tyne, England, United Kingdom; Newcastle Univesrity, Newcastle Upon Tyne, England, United Kingdom; Newcastle University, Newcastle upon Tyne, England, United Kingdom; Newcastle University, Newcastle upon Tyne, England, United Kingdom

## Abstract

Older people in the UK experience the highest rates of alcohol-related harm of all age groups, due to reduced tolerance to alcohol with age. Most older people drinking at harmful levels have co-occurring mental health problems. This population fall between services, which are ill-equipped to address their complex and multifaceted needs. This study aimed to co-design directions for integrated services to support this group in North East/North Cumbria, England. Co-design was guided by Intervention Mapping, and was informed by four workshops with older people with co-occurring alcohol/mental health problems; informal caregivers and supporting practitioners. Workshops built on evidence from i) a preceding qualitative study with these groups examining lived experience, support needs, and challenges in supporting this group and ii) a literature review of existing interventions/services for older people with alcohol or mental health problems. Findings highlight the importance of availability, accessibility and flexibility of support for older people, who often present in crisis, and can face additional barriers to accessing services such as hearing/mobility issues. Mental health practitioners should be trained in identifying and addressing harmful drinking in older people. Practitioners must be knowledgeable of relevant, local services and prepared to signpost appropriately. Care should encompass peer support, and support and advocacy to address wider issues that impact mental health and alcohol use including finances, social isolation and illness. This study informs the development of integrated community alcohol/mental health support for this patient group. Applicability of directions to other care systems is considered.

